# Willingness to receive the COVID-19 vaccine among HIV positive men who have sex with men in China: a cross-sectional study

**DOI:** 10.1186/s12889-022-14961-5

**Published:** 2023-01-10

**Authors:** Songjie Wu, Shanhui Zhu, Xumeng Yan, Yongshi Xu, Huifang Xu, Fang Yang, Zhigang Han, Yuzhou Gu, Yi Zhou, Zhengrong Yang, Huake Yang, Bo Shu, Weiming Tang, Ke Liang

**Affiliations:** 1 Department of Nosocomial Infection, Zhongnan Hospital of Wuhan University, Wuhan, China; 2 Wuhan Research Center for Infectious Diseases and Cancer, Chinese Academy of Medical Sciences, Wuhan, 430071 China; 3 Medical Department, Zhongnan Hospital of Wuhan University, Wuhan, China; 4 University of North Carolina at Chapel Hill Project-China, Guangzhou, China; 5 Department of HIV/AIDS Control and Prevention, Guangdong Provincial Center for Disease Control and Prevention, Guangzhou, China; 6 Department of HIV/AIDS Control and Prevention, Guangzhou Center for Disease Control and Prevention, Guangzhou, China; 7 Department of HIV/AIDS Control and Prevention, Zhuhai Center for Disease Control and Prevention, Zhuhai, China; 8 Department of HIV/AIDS Control and Prevention, Shenzhen Center for Disease Control and Prevention, Shenzhen, China; 9 Department of HIV/AIDS Control and Prevention, Dongguan Center for Disease Control and Prevention, Dongguan, China; 10 Department of HIV/AIDS Control and Prevention, Zhongshan Center for Disease Control and Prevention, Zhongshan, China; 11 Department of Infectious Diseases, Zhongnan Hospital of Wuhan University, Wuhan University, Hubei, China; 12 Hubei Engineering Center for Infectious Disease Prevention, Control and Treatment, Wuhan, China

**Keywords:** COVID-19, Vaccine, Willingness, HIV, MSM

## Abstract

**Background:**

People living with HIV(PLWH) are deemed more vulnerable to the SARS-CoV-2 infection than the uninfected population. Vaccination is an effective measure for COVID-19 control, yet, little knowledge exists about the willingness of men who have sex with men (MSM) living with HIV in China to be vaccinated.

**Methods:**

This cross-sectional study evaluated the willingness of MSM living with HIV to receive COVID-19 vaccination in six cities of Guangdong, China, from July to September 2020. Factors associated with willingness to receive COVID-19 vaccination using multivariable logistic regression.

**Results:**

In total, we recruited 944 HIV-positive MSM with a mean age of 29.2 ± 7.7 years. Of all participants, 92.4% of them were willing to receive the COVID-19 vaccine. Participants who were separated, divorced, or widowed (adjusted OR: 5.29, 95%CI: 1.02–27.48), had an annual income higher than 9,000 USD (adjusted OR: 1.70, 95%CI: 1.01–2.86), had ever taken an HIV self-test (adjusted OR: 1.78, 95%CI: 1.07–2.95), had ever disclosed sexual orientation to a doctor/nurse (adjusted OR: 3.16, 95%CI: 1.33–7.50), had ever disclosed sexual orientation to others besides their male partners (adjusted OR: 2.18, 95%CI: 1.29–3.69) were more willing to receive the vaccine. Sex with a female partner in the past six months decreased the likelihood of willingness to receive the vaccine (adjusted OR: 0.40, 95%CI: 0.17–0.95). Economic burden, worry that my health condition could not bear the risk of receiving COVID-19 vaccines, and concern that the vaccination would affect the immune status and antiretroviral therapy were the main reasons for unwillingness to receive vaccination.

**Conclusion:**

Our study showed that HIV-positive MSM had a high willingness to receive the COVID-19 vaccination. Targeted interventions such as health education should be conducted among MSM with HIV infection to enhance COVID-19 vaccine uptake.

## Introduction

The coronavirus disease 2019 (COVID-19) is a severe respiratory infection caused by the severe acute respiratory syndrome coronavirus 2 (SARS-CoV-2). The pandemic reportedly caused more than 500 million infections and 6 million deaths globally by May 2022 [1]. Studies have shown that people living with HIV(PLWH) have an increased risk of SARS-CoV-2 infection and mortality [2]. The COVID-19 pandemic also brought other impacts to PLWH, including affecting their physical, emotional, and social well-being, interfering with the delivery of effective healthcare and access to HIV treatment [3]. Surveillance data had shown that over one-third of men who have sex with men (MSM) living with HIV in the United States were not on adequate treatment and may be immunocompromised, potentially increasing risks associated with susceptibility of COVID-19 [4]. And the stigma and discrimination for MSM also may result in health inequities, which bring the higher risk for COVID-19 [5, 6].

Vaccination is considered one of the most effective strategies for infectious diseases prevention and control [7]. Existing surveys suggest that the COVID-19 vaccination should reach a minimum coverage of 70% to achieve the herd immunity required for epidemic control [8]. Several COVID-19 vaccines have been approved for use in many countries [9]. However, the vaccination uptake was still low [10], especially among the vulnerable population [11, 12]. Improved understanding of the willingness to receive COVID-19 vaccines and associated factors can inform more comprehensive and equitable vaccine implementation strategies [13]. Several studies have explored the willingness to receive COVID-19 vaccination and its associated factors among different populations. In the general population, the rates of COVID-19 vaccination willingness reportedly range from 23.6 to 97% globally [14–16] and higher than 80% in China [17, 18]. Furthermore, a previous study among French PLWH showed that approximately 70% were willing to receive the COVID-19 vaccine and others hesitated for various reasons [19]. Another study conducted in the USA reported a moderately high acceptance of the COVID-19 vaccine among sexual and gender minority men and transgender women [20]. However, studies assessing willingness to accept COVID-19 vaccination among HIV-positive MSM in low- and middle-income countries, including China, are rare.

In China, seven COVID-19 candidate vaccines are in clinical trials, three of which are in Phase 3 trials as of August 2020 [21]. The results showed that the candidate COVID-19 vaccines had good immunogenicity and safety [21]. Even though the COVID-19 epidemic has slowed [22], multiple local outbreaks in China along with the ongoing import of cases from overseas. This put the vast majority of the Chinese population without immunity against SARS-CoV-2, especially vulnerable groups like PLWH, at continued risk of infection. In response to the potential re-emergence of COVID-19 epidemics in China, it is necessary to understand the willingness of MSM living with HIV to be vaccinated and take measures to improve vaccination coverage.

Till now, little is known about the willingness to receive COVID-19 vaccination among HIV-positive MSM and associated factors in China, which bring difficulty for controlling the pandemic of COVID-19 among people living with HIV. This study aimed to investigate the willingness to receive the COVID-19 vaccine and to assess the factors associated with COVID-19 vaccination among HIV-positive MSM to provide a reference for COVID-19 vaccine promotion strategies. We hypothesized that factors associated with the COVID-19 vaccination willingness among HIV-positive MSM were various and perceptions of COVID-19 vaccination would affect their willingness.

## Methods

### Study design and participants

This cross-sectional study was performed between July and September 2020. Study participants complied with the following inclusion criteria: born as male; ≥ 18 years old; identified as living with HIV in the past two years; transmitted through homosexual route; and currently live in the six southern cities (Guangzhou, Shenzhen, Zhongshan, Dongguan, Foshan, and Zhuhai) of Guangdong Province, China. Participants who were not able to understand the questionnaire items or refused to be surveyed were excluded. All the participants were recruited by a convenience sampling technique through sending an invitation to the registered MSM HIV cases identified after 2018 by the local Center for Disease Control and Prevention (CDC) or the HIV healthcare centers. After the screening, all the eligible participants electrically signed on the informed consent and completed an online self-administered questionnaire. The Chinese online survey tool, Wenjuanxing (www.wjx.cn), was used to administer the survey. The sample size was computed via the formula N = Z_α_^2^ *p* (1—*p*)/d^2^, where α = 0.05 and Z_α_ = 1.96. The estimated acceptable margin of error for proportion d was 0.05, and the proportion of MSM living with HIV who were willing to receive the COVID-19 vaccination was estimated at 50%. Finally, the minimum sample size was estimated at about 385. This proposed study aimed to recruit 1000 newly identified HIV-positive MSM for an HIV partner notification survey across the six study cities in Guangdong province of China [23], which is more than the minimum sample size required.

### Measures

Sociodemographic information, including age, employment status (employed or unemployed), marital status (unmarried, engaged or married, separated or divorced, widowed), education level (high school or lower, higher than high school), annual income (≤ 9000USD, > 9000USD), gender identity (male, female, transgender, unsure/other), and sexual orientation (homosexual, bisexual, heterosexual, unsure/other), was collected from each eligible participant. Solicited data on sexual behavior in the preceding six months included ever had sex with a female partner, ever had multiple male sexual partners, ever had stable male sex partners, ever had casual male sex partners, and so on. Clinical characteristics assessed included whether participants had comorbidities, had ever self-tested for HIV, the first CD4 + T lymphocyte count (CD4 count). Sexual orientation disclosure, defined as having discussed their sexual orientation with a doctor/nurse or others, was also assessed. The participants’ perception of susceptibility was also collected, which was measured by a single yes or no item: "Do you think HIV-positive members are more likely to get infected with COVID-19?". Willingness to receive the COVID-19 vaccination was measured by the single yes or no item "Would you be willing to receive COVID-19 vaccine if provided?". The reasons for willingness or unwillingness to receive the COVID-19 vaccination was also asked by some yes or no questions (multiple answers).

### Statistical analysis

Categorical variables were presented as numbers (n)/percentage (%) and compared with the Chi-square test or Fisher's exact test. Continuous variables were presented as mean and standard deviation (normally distributed) or the median and interquartile range (non-normally distributed) and compared with the t-test or Wilcoxon rank-sum test. Univariable and multivariable logistic regression analysis was used to determine whether any factors (age groups, educational level, annual income, employment status, and other factors) were associated with the willingness of COVID-19 vaccination. A stepwise forward method of selecting variables was applied and variables with *p* values < 0.10 in the likelihood ratio test entered the multivariable logistic regression model. The goodness of fit to the logistic regression model was tested using the Hosmer–Lemeshow goodness-of-fit test. The associations between the potential influencing factors and outcomes were presented as odds ratios (ORs) and 95% confidence intervals (CIs). A two-tailed *p*-value < 0.05 was statistically significant. Data were analyzed using SPSS version 26 (IBM Corp., Armonk, NY).

### Ethics statement

This study was approved by Ethics Committee of Guangzhou Center for Disease Control and Prevention (GZCDC-ER-P2019001). Signed electronic informed consent forms were obtained from all participants involved in the study.

## Results

### Characteristics of study participants

Total 944 participants were recruited in this study. The mean age of the participants was 29.2 ± 7.7 years. The majority of participants were unmarried (81.9%), employed (96.9%), had higher than high school education (54.5%), and 53.2% (502/944) earned 9000 USD or less annually. 95.1% (898/944) had self-identified their gender as male, and 70.8% (668/944) were homosexuals (Table 1).

**Table 1:** Descriptive characteristics of MSM who are living with HIV in six cities of Guangdong, China, 2020 (*N* = 944)

Item	Total	Willing to receive the COVID-19 vaccine(n,%)	Unwilling to receive the COVID-19 vaccine(n,%)	P
**Sociodemographics**				
Age (Mean ± SD)	29.2 ± 7.7	29.2 ± 7.6	28.6 ± 8.1	0.53
≤ 29	570	526(92.3)	44(7.7)	
30–39	272	251(92.3)	21(7.7)	
≥ 40	102	95(93.1)	7(6.9)	0.95
Employment status				
Employed	915	847(92.6)	68(7.4)	
Unemployed	29	25(86.2)	4(13.8)	0.20
Marital status				
Unmarried	773	712(92.1)	61(7.9)	
Engaged or Married	84	75(89.3)	9(10.7)	
Separated, Divorced or Widowed	87	85(97.7)	2(2.3)	0.09
Educational level				
High school or lower	429	388(90.4)	41(9.6)	
Higher than high school	515	484(94.0)	31(6.0)	0.04
Annual income				
≤ 9000 USD	502	456(90.8)	46(9.2)	
> 9000 USD	442	416(94.1)	26(5.9)	0.06
Gender identify				
Male	898	831(92.5)	67(7.5)	
Female	8	8(100.0)	0(0.0)	
Transgender	9	7(77.8)	2(22.2)	
Unsure/Other	29	26(89.7)	3(10.3)	0.29
Sexual orientation				
Homosexual	668	620(92.8)	48(7.2)	
Bisexual	220	203(92.3)	17(7.7)	
Others	56	49(87.5)	7(12.5)	0.41
**Perception of susceptibility**				
HIV positive members are more likely to get infected with Covid-19				
Yes	151	140(92.7)	11(7.3)	
No	210	191(91.0)	19(9.0)	
Not sure	583	541(92.8)	42(7.2)	0.68
**Clinical characteristics**				
Have comorbidities				
No	698	646(92.5)	52(7.5)	
Yes	113	105(92.9)	8(7.1)	
Not sure	133	121(91.0)	12(9.0)	0.80
The first CD4 count				
< 500	418	388(92.8)	30(7.2)	
≥ 500	362	335(92.5)	27(7.5)	
Not sure/don’t remember /not tested	164	149(90.8)	15(9.2)	0.72
**Sexual behavior**				
Had ever taken an HIV self-test				
Yes	558	525(94.1)	33(5.9)	
No	386	347(89.9)	39(10.1)	0.02
Had multiple male sexual partners in the last six months				
Yes	255	229(89.8)	26(10.2)	
No	689	643(93.3)	46(6.7)	0.07
Had sex with a female partner in the past six months				
Yes	50	41(82.0)	9(18.0)	
No	894	831(93.0)	63(7.0)	0.005
Had stable male sex partner(s) in the past six months				
Yes	327	305(93.3)	22(6.7)	
No	617	567(91.9)	50(8.1)	0.58
Had casual male sex partner(s) in the past six months				
Yes	241	220(91.3)	21(8.7)	
No	703	652(92.7)	51(7.3)	0.64
**Sexual Orientation Disclosure**				
Ever disclosed to a doctor/nurse				
Yes	217	211(97.2)	6(2.8)	
No	727	661(90.9)	66(9.1)	0.002
Ever disclosed to others besides their male partners				
Yes	782	736(94.1)	46(5.9)	
No	162	136(84.0)	26(16.0)	< 0.001

Among them, 26.1% (246/944) had comorbidities (hypertension, diabetes, or others), 44.3% (418/944) had the first CD4 count lower than 500/μL, and 59.1% (558/944) had ever taken an HIV self-test; 73.0% (689/944) did not have multiple male sexual partners in the last six months, 94.7%(894/944) did not have sex with a female partner in the past six months, 34.6% (327/944) had stable male sex partners in the past six months, and 25.5% had casual male sex partners in the past six months. Besides, 23.0% (217/944) had ever disclosed their sexual orientation to a doctor/nurse, and 82.8% (782/944) had ever disclosed their sexual orientation to others besides their male partners.

### Willingness to receive the COVID-19 vaccine

**Fig. 1:**
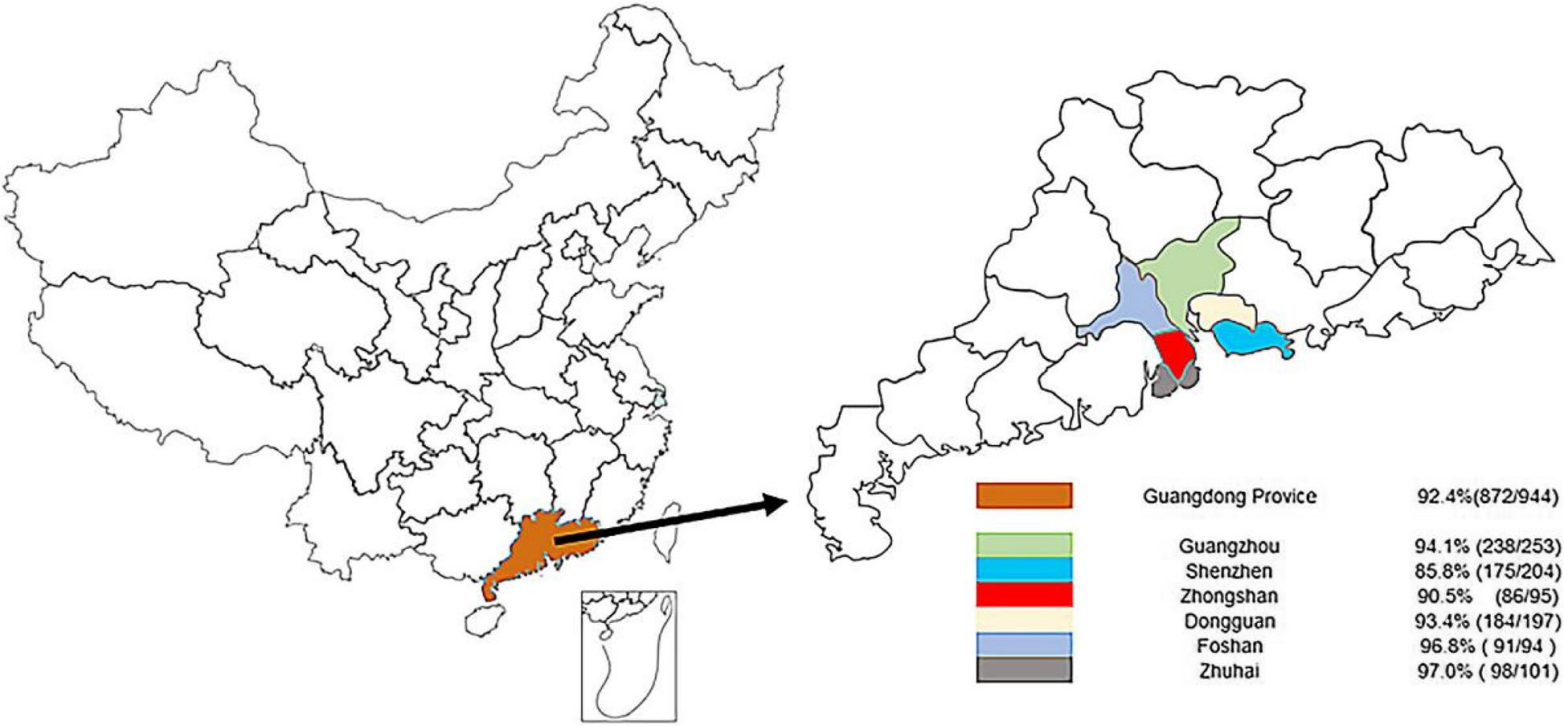
Willingness to receive COVID-19 vaccination in different cities

The willingness to receive COVID-19 vaccination in different cities were shown in Fig. 1. Overall, 92.4% (872/994) of participants were willing to receive the COVID-19 vaccine, and 60.3% (569/944) were willing to pay for the COVID-19 vaccine.

A significantly higher willingness was observed in MSM living with HIV who had high school level or higher education, had ever self-tested for HIV, disclosed their sexual orientation to a doctor/nurse, disclosed their sexual orientation to others besides their male partners and had not have sex with a female partner in the past six months (all *p* < 0.05) (Table 1).

### Factors associated with willingness to receive the COVID-19 vaccine

Univariate logistic regression showed that marital status, educational level, ever had taken an HIV self-test, sexual orientation disclosure to a doctor/nurse, sexual orientation disclosure to others besides male partners and ever had sex with a female partner in the past six months were associated with willingness to receive COVID-19 vaccination among MSM living with HIV (*p* < 0.05, unadjusted ORs were shown in Table 2).

**Table 2:** Univariable and multivariable logistic regression analysis showing the factors associated with the willingness of MSM living with HIV in China to receive the COVID-19 vaccine (*n* = 944)

Item	Univariable logistic regression		Multivariable logistic regression	
	Unadjusted OR (95%CI)	P	Adjusted OR (95%CI)	P
**Sociodemographic**				
Age (Mean ± SD)	1.01(0.98–1.04)	0.53		
≤ 29	ref			
30–39	1.00(0.58–1.72)	0.99		
≥ 40	1.14(0.50–2.60)	0.76		
Employment status				
Employed	ref			
Unemployed	0.50(0.17–1.48)	0.21		
Marital status				
Unmarried	1.401(0.67–2.93)	0.37	1.08(0.39–2.97)	0.88
Engaged or Married	ref		ref	
Separated or Divorced or Widowed	5.10(1.07–24.35)	0.04	5.29(1.02**–27.48)**	< 0.05
Educational level				
High school or lower	ref			
VHigher than high school	1.65(1.02–2.68)	0.04		
Annual income				
≤ 9000 USD	ref		ref	
> 9000 USD	1.61(0.98–2.66)	0.06	1.70(1.01–2.86)	< 0.05
Gender identify				
Male	ref			
Female	-	0.99		
Transgender	0.28(0.06–1.39)	0.12		
Unsure/Other	0.70(0.21–2.37)	0.57		
Sexual orientation				
Homosexual	ref			
Bisexual	0.92(0.52–1.64)	0.79		
Others	0.54(0.23–1.26)	0.16		
**Perception of susceptibility**				
HIV positive members are more likely to get infected with Covid-19				
No	ref			
Yes	1.27(0.58–2.75)	0.55		
Not sure	1.28(0.73–2.26)	0.39		
**Clinical characteristics**				
Have comorbidities				
No	ref			
Yes	1.06(0.49–2.29)	0.89		
Not sure	0.81(0.42–1.57)	0.53		
First CD4 count				
< 500	ref			
≥ 500	1.04(0.61–1.79)	0.88		
Not sure/don’t remember /not test	1.30(0.68–2.49)	0.43		
**Sexual behavior**				
Had ever taken an HIV self-test				
No	ref			
Yes	1.79(1.10–2.90)	0.02	1.78(1.07–2.95**)**	0.03
Had multiple male sexual partners in the last six months				
No	ref			
Yes	0.63(0.38–1.04)	0.07		
Had sex with a female partner in the past six months				
No	ref			
Yes	0.35(0.16–0.74)	< 0.05	0.40(**0.**17**–**0.95)	0.04
Had stable male sex partner(s) in the past six months				
No	ref			
Yes	1.16(0.69–1.94)	0.577		
Had casual male sex partner(s) in the past six months				
No	ref			
Yes	0.82(0.48–1.39)	0.462		
**Sexual orientation disclosure**				
Ever disclosed to a doctor/nurse				
No	ref			
Yes	3.51(1.50–8.22)	< 0.05	3.16(1.33–7.50)	< 0.05
Ever disclosed to others besides their male partners				
No	ref			
Yes	2.36(1.42–3.90)	< 0.05	2.18(1.29–3.69)	< 0.05

We further adjusted confounding factors using a forward stepwise multivariable logistic regression model, the Nagelkerke R^2^ showed that the model is explaining 14.4% of the variation in willingness to receive COVID-19 vaccination, the outcome of Hosmer and Lemeshow Test (χ2 = 9.87, df = 7, *p* = 0.196) showed the model had a good fit. According to the results of Table 2, participants who were separated, divorced, or widowed (adjusted OR: 5.29, 95%CI: 1.02–27.48), had an annual income higher than 9000 USD (adjusted OR: 1.70, 95%CI: 1.01–2.86), had ever taken an HIV self-test (adjusted OR: 1.78, 95%CI: 1.07–2.95), sexual orientation disclosure to a doctor/nurse (adjusted OR: 3.16, 95%CI: 1.33–7.50), and sexual orientation disclosure to others besides male partners (adjusted OR: 2.18, 95%CI: 1.29–3.69) were associated with increased likelihood of willingness to receive COVID-19 vaccination among MSM living with HIV. However, participants who had ever had sex with a female partner in the past six months were associated with decreased the likelihood of willingness to receive the vaccine (adjusted OR: 0.40, 95%CI: 0.17–0.95).

### Reasons for vaccination decisions

**Fig. 2:**
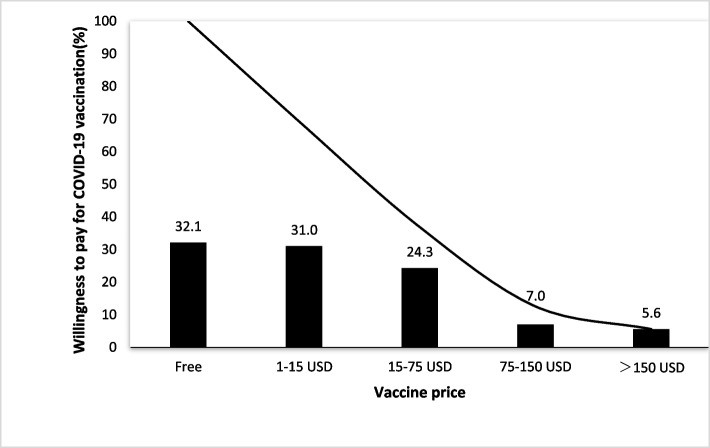
Willingness to pay for COVID-19 vaccination

As shown in Fig. 2, 32.1% (280/872) participants cited a willingness to be vaccinated when provided for free. We observed a J-shaped distribution of the willingness to accept vaccination among willing participants, and 30.0% (270/872) of participants expressed their willingness to pay below 15 USD, which declined to 5.6% (49/872) when the price was higher than 150 USD.

The main reasons for unwillingness to receive the COVID-19 vaccine were “economic burden (43.1%)”, “Worry that my health condition could not bear the risk of receiving COVID-19 vaccines (30.6%)”, “Worry that the vaccination will affect my immune status and antiretroviral therapy” and “Worry about the quality of the vaccine” (22.2%). Moreover, the main reasons for willingness to be vaccinated were “I don’t want to get infection with SARS-CoV-2 (78.4%)”, "I worry that my risk of COVID-19 infection is higher than normal people (47.5%)”, and “I worry that my condition will be worse than normal people if infected (37.6%)” (Table 3).

**Table 3:** Reasons for vaccination decisions of MSM who are living with HIV in six cities of Guangdong, China, 2020

Item	N (%)
**Reasons for unwillingness to receive the COVID-19 vaccine (*N* = 72)**	
Economic burden	31 (43.1)
Worry that my health condition could not bear the risk of receiving COVID-19 vaccines	22 (30.6)
Worry that the vaccination will affect my immune status and antiviral therapy	20(27.8)
Worry about the quality of the vaccine	16 (22.2)
I don’t like receiving vaccines	13 (18.1)
I don’t think COVID-19 is serious	8 (11.1)
It is not the most urgent health need	7 (9.7)
I am willing to take the risk of COVID-19 infection	1(1.4)
Other	2(2.8)
**Reasons for willingness to receive the COVID-19 vaccine (*N* = 872)**	
I don’t want to get infected with COVID-19	684(78.4)
I worry that my risk of COVID-19 infection is higher than normal people	414(47.5)
I worry that my condition will be worse than normal people if infected	328(37.6)
I believe the vaccine can help prevent infection	320(36.7)
I believe the vaccine is safe in most cases	132(15.1)
I am used to receiving vaccines	67(7.7)
Other	9(1.0)

## Discussion

Understanding the willingness to receive the COVID-19 vaccine among HIV-positive MSM is essential in improving the COVID-19 vaccination rate. We found that the majority of the MSM living with HIV were willing to receive the COVID-19 vaccine. Factors including having ever taken an HIV self-test, sexual orientation disclosure to a doctor/nurse or others, annual income, and marital status were associated with COVID-19 vaccination willingness.

Our study showed that around 92% of the HIV-positive MSM are willing to receive the COVID-19 vaccine. This proportion was higher than that of PLWH in France [19] and the United States [20] as well as the general population in most parts of the world [24–26] and China [17]. Findings from several studies suggested that the willingness to get vaccinated against COVID-19 has evolved [27]. Even though willingness was relatively high in this study, we should continually be concerned about the willingness to get vaccination against COVID-19 among HIV-positive MSM at different times.

In our study, the participant who had ever disclosed their sexual orientation to a doctor/nurse or others besides their male partners and those who ever self-tested for HIV had a higher willingness to receive the COVID-19 vaccine. Studies have shown that HIV-positive MSM who had ever disclosed their sexual orientation to health providers or ever self-tested for HIV had less HIV stigma [28], while HIV stigma was a barrier for accessing health services, including vaccinations [29]. Previous studies have also shown the introduction of quality comprehensive HIV care to reduce stigma and increase uptake of HIV services, including vaccinations [30, 31]. MSM living with HIV who had ever disclosed their sexual orientation to a nurse/doctor were more likely to receive the COVID-19 vaccine, which suggests that the health providers could play an essential role in promoting the uptake of COVID-19 vaccines. Also, reports show that MSM with full disclosure to a primary clinician may have increased optimal uptake of vaccinations [32]. Thus, potential interventions to promote sexual orientation disclosure without prejudice may help to improve COVID-19 vaccination uptake [32]. In addition, participants who had ever self-tested for HIV may have better knowledge and awareness about HIV [33], and that may cause them to have a higher willingness for the vaccine.

Our study findings also suggest that separated, divorced, or widowed MSM living with HIV had a higher likelihood of willingness to receive COVID-19 vaccination than married ones. This finding was inconsistent with other studies [17, 34]. Separated, divorced, or widowed living alone with limited assistance often could have accounted for this finding. They may have limited access to healthcare, irregular health facility visits, and less support from family members [35]. And this may cause them to seek preventive measures that protect them from further infections. Our study also showed that MSM living with HIV who earned more than 9000 USD annually had a higher willingness to receive the COVID-19 vaccine. This observation is similar to the results of some other studies [36, 37] and could be because people with higher income may pay more attention to their health and adopt healthier COVID-19 prevention behaviors. Besides, the rate of willingness to receive COVID-19 vaccination declined from 92.4 to 62.1% if the vaccine would not be free, and only a few participants (2.5%) would afford more than 150 USD for it. Our study results showed that participants perceive ‘Economic burden’ as a significant barrier to receive COVID-19 vaccination. The results also suggest that offering the COVID-19 vaccines for a lower cost or free might improve the willingness to receive vaccination among MSM living with HIV. While this may not be a barrier in China as COVID-19 vaccinations are currently offered for free [38] but may hinder uptake in countries with paid COVID-19 vaccination programs.

Our study showed different reasons for willingness or unwillingness to receive the COVID-19 vaccine. The main reasons for unwillingness to receive the COVID-19 vaccination indicate that participants may have concerns about the side effects and the impact on the immune status and antiviral therapy after the COVID-19 vaccination. However, the United Nations AIDS program (UNAIDS) recommends that COVID-19 vaccines are safe for PLWH and could bring them the same benefits as uninfected individuals. Hence, the COVID-19 vaccination should be recommended for PLWH regardless of their CD4 count and HIV viral load levels and possibly prioritized in vaccination rollouts [39]. The national technical guideline for COVID-19 vaccination in China encourages PLWH to take up inactivated vaccines or recombinant subunit vaccines [40]. A recent pilot study conducted by the Chinese National Center for Clinical Laboratories found that 60 and 63% of PLWHA who had received two doses of COVID-19 vaccine stimulate neutralizing antibody or T-cell responses, respectively, without any severe side effect after vaccination [41]. Therefore, perceptions on COVID-19 vaccination should be improved among HIV-positive MSM.

This study provided crucial information on the willingness to receive COVID-19 vaccination among HIV-positive MSM and associated factors, which could provided reference for developing tailored vaccination strategies for HIV-positive MSM. More attention should be paid to HIV-positive MSM who had lower income as these group reported lower willingness. Additionally, health education programs should be designed and developed to improve perceptions related to COVID-19 vaccination among HIV-positive MSM and their confidence in COVID-19 vaccines to improve the vaccination rate.

Some of the advantages of our research are worth noticing. This study was investigated 4 months before the initiation of national free vaccination policy, which offered the initial insights about willingness to receive COVID-19 vaccination among HIV-positive MSM with relatively large sample. The study also had some limitations. First, information on sexual behaviors was collected using a questionnaire. Therefore, sexual risks may have been over-reported or under-reported due to reporting and social desirability bias. However, administering the questionnaire online provided anonymity that reduced the risk of this bias. Second, our study participants might not represent the general population of MSM living with HIV in China due to the potential limitations of our purposive enrollment methods. Third, the cross-sectional study method limited association analysis. Fourth, our study was conducted before September 2020, when the COVID-19 vaccine had not been approved in China yet. Hence, it was unknown whether the COVID-19 vaccines would be offered freely in China. Moreover, the limited information about vaccination eligibility and locations influenced people’s thoughts, decisions, and perceptions about getting vaccinated. Therefore, an individual’s willingness to get vaccinated may evolve with new information [42, 43]. However, our findings showed strong demand for the vaccine and the high recognition of vaccination importance among MSM with HIV. And this could still provide a reference for the implementation of vaccination programs among MSM living with HIV. Despite the above limitations, our study has important strengths. We examined the willingness to receive COVID-19 vaccination using a large-scale sample of participants. We would subsequently conduct a more comprehensive research at a different time to compare with the results of this study next.

## Conclusions

In conclusion, our study showed that a large majority of MSM living with HIV are willing to receive the COVID-19 vaccine. MSM living with HIV in China who had ever taken an HIV self-test and disclosed their sexual orientation to a doctor/nurse or others were more willing to receive the COVID-19 vaccination. Targeted interventions such as health education should be conducted among MSM with HIV infection to enhance COVID-19 vaccine uptake.

## Data Availability

The datasets used and/or analyzed during the current study are not publicly available due to protect the privacy and confidentiality of participants in this study but are available upon reasonable request to the corresponding author.

## Abbreviations

PLWH: People living with HIV
MSM: Men who have sex with men
SARS-CoV-2: Severe acute respiratory syndrome coronavirus 2
COVID-19: Coronavirus disease 2019
CDC: Center for Disease Control and Prevention
Cis: Confidence intervals
